# International Criteria for Acute Kidney Injury: Advantages and Remaining Challenges

**DOI:** 10.1371/journal.pmed.1002122

**Published:** 2016-09-13

**Authors:** Nicholas M. Selby, Richard J. Fluck, Nitin V. Kolhe, Maarten W. Taal

**Affiliations:** 1 Centre for Kidney Research and Innovation, Division of Medical Sciences and Graduate Entry Medicine, University of Nottingham, Royal Derby Hospital Campus, Derby, United Kingdom; 2 Department of Renal Medicine, Royal Derby Hospital, Derby, United Kingdom

## Abstract

Nicholas Selby and colleagues describe how the definition of acute kidney injury brings opportunities and challenges in identifying patients at higher risk of adverse outcomes.

**Summary Points**

- Acute Kidney Injury (AKI) is defined using widely accepted international criteria that are based on changes in serum creatinine concentration and degree of oliguria.
- AKI, when defined in this way, has a strong association with poor patient outcomes, including high mortality rates and longer hospital admissions with increased resource utilisation and subsequent chronic kidney disease.
- The detection of AKI using current criteria can assist with AKI diagnosis and stratification of individual patient risk.
- The diagnosis of AKI requires clinical judgement to integrate the definition of AKI with the clinical situation, to determine underlying cause of AKI, and to take account of factors that may affect performance of current definitions.

## Introduction

Acute kidney injury (AKI), previously termed acute renal failure, has been the focus of increasing attention in the medical and popular press because of its high incidence and strong association with poor patient outcomes. Alarming figures include an incidence of between 5%–22% of hospital admissions and mortality rates that exceed 50% in those most severely affected [1–4]. The impact of AKI in low- and middle-income countries (LMICs) is equally stark: estimates of the global burden of AKI suggest that over 13 million people are affected annually and that AKI contributes to 1.7 million deaths per year [5]. There is also growing appreciation that renal function does not always recover following AKI and that AKI and chronic kidney disease (CKD) have a bidirectional relationship, with each increasing the risk of the other [6,7]. Such long-term health consequences coupled with prolonged hospital stays and considerable health care costs emphasise the huge societal impact of AKI [8,9]. These statistics have led to a number of calls to increase awareness of AKI and allocate more resources towards its prevention and treatment, including the International Society of Nephrology’s 0by25 campaign that aims to eliminate all preventable deaths related to AKI by 2025 [10,11].

## Diagnosing Acute Kidney Injury

In part, increased awareness has sprung from the widespread adoption of international criteria for the definition of AKI that are based on changes in serum creatinine concentration and degree of oliguria (Table 1) [12–14]. These criteria include small changes in serum creatinine alongside larger increments and receipt of renal replacement therapy (RRT). AKI therefore represents a wide spectrum of patients in comparison to historical concepts of acute renal failure. There is no doubt that this harmonisation in AKI definition has been a major step forward, replacing more than 35 previously used definitions in the medical literature [15] and focussing attention on opportunities of prevention and recognition that arise earlier in the natural history of AKI. A standardised definition is essential for robust epidemiological research to define the magnitude of the problem posed by AKI; a clear association between AKI defined in this way and patient outcomes has been consistently shown across a large number of studies [3,16]. The magnitude of this association is strong (e.g., odds ratios for mortality in the range of 5–10, [17]), is proportional to the severity of AKI, and remains remarkably consistent across every clinical condition and environment in which it has been studied. Similar high mortality and associations of AKI severity with outcomes are also seen in data from LMICs, despite the relative paucity of studies and the many differences in AKI epidemiology in these settings [18]. These findings have been extremely important in highlighting the challenges posed by AKI much more widely, as is necessary with the majority of AKI care delivered by non-nephrologists [1]. AKI has now become a term that is widely understood in clinical discussions, and the criteria provide a structure for education and guidelines, describing the severity of AKI as well as just its presence. Whilst a standardised definition brings advantages, it also brings challenges when applied to the syndrome of AKI to clinical practice. Although some may take these challenges as reason not to use current AKI classification outside of epidemiological research, we would argue that such an approach would restrict the benefits that a standardised definition of AKI brings. Here, we discuss the practical considerations that are essential to consider when doing so.

**Table 1:** KDIGO (Kidney Disease: Improving Global Outcomes) criteria for classification of acute kidney injury [14]. Both serum creatinine and urine output criteria should be applied, and the AKI stage should be taken as whichever is the higher.

**AKI Stage**	**Serum Creatinine Increase**	**Urine Output**
1	1.5–1.9 times baseline OR ≥26.5 μmol/L (0.3 mg/dl) increase within 48 hours	<0.5 ml/kg/hour for 6–12 hours
2	2.0–2.9 times baseline	<0.5 ml/kg/hour for ≥12 hours
3	3.0 times baseline OR increase in serum creatinine to ≥353.6 μmol/L (4.0 mg/dl)OR start renal replacement therapy	<0.3 ml/kg/hour for ≥24 hoursOR anuria for ≥12 hours

## Challenges of Applying AKI Diagnostic Criteria in Clinical Practice

### Aetiology, Baseline Creatinine, and Preexisting CKD

Classifying a patient with AKI should not detract from identification of the underlying cause; whilst it may be obvious that AKI is heterogeneous and that different aetiologies require distinct therapeutic approaches, AKI aetiology is not part of current AKI definitions. Use of a standard definition may therefore create the false impression that all cases of AKI are similar and can be managed in the same way, which must be avoided. This argument can be extended to include consideration of the clinical context in which AKI occurs and its relationship to outcomes: for example, AKI in the setting of sepsis worsens prognosis; urological AKI (with a higher proportion of obstructive causes) has the opposite effect [19].

As AKI is defined on an individual basis with respect to baseline serum creatinine value, choice of different baseline values can have a significant effect on the sensitivity and specificity of AKI criteria [20,21]. In addition, a baseline serum creatinine may not always be available in clinical practice. On a case-by-case basis, this can usually be resolved by clinician interpretation of serial creatinine results or an early repeat measurement to determine rate of change. The issue has more relevance to database studies or the development of electronic detection and alerting systems, where there is a need for greater standardisation of definitions for baseline creatinine [22,23].

Interpreting AKI criteria can also be more challenging in patients with preexisting CKD. Higher baseline creatinine values mean that small changes (e.g., rises of 26.5 μmol/l or 0.3 mg/dl) in serum creatinine concentration might represent laboratory or biological variation rather than a true change in renal function [24]; conversely, reduced renal reserve in individuals with CKD confers increased vulnerability [25]. Furthermore, the combination of a threshold value and percentage changes in serum creatinine to describe AKI stage 3 somewhat reduces the gradated association of AKI severity with mortality in those with higher baseline serum creatinine values [1,20]. Whilst some have advocated alternate classifications of AKI using only absolute changes in serum creatinine, there is insufficient evidence to recommend such approaches currently.

### Should Small Creatinine Changes Be Included?

There have been concerns from some quarters that the inclusion of relatively small increases in serum creatinine may result in overdiagnosis, labelling patients with AKI who actually have clinically insignificant biochemical changes and risking exposure to at best unnecessary and at worst potentially harmful treatment [26]. It is true that small changes in serum creatinine concentration may not always represent clinically meaningful reductions in glomerular filtration rate (GFR). There is both biological and laboratory variation in serum creatinine measurements; the former may result from changes in diet, creatinine generation, volume status, tubular secretion, and actions of certain medications, whilst the latter is more pronounced in patients with CKD and high baseline creatinine values [24]. However, inclusion of these smaller serum creatinine changes reflects their strong epidemiological associations with adverse outcomes, including mortality and long-term risk of CKD that persist even after adjustment for age and comorbidities [17,27–34]. There are also data to show that the inclusion of small creatinine changes improves early diagnosis of AKI compared to previous criteria [35], in combination with education programmes [36] or if used to generate electronic alerts [37]. Small creatinine changes should not be ignored but rather integrated with the clinical picture, not only for AKI diagnosis but also to help gauge overall patient risk. There are examples of the latter: combining clinical risk factors for AKI with small creatinine changes produced a good predictive model for more severe AKI (stages 2 or 3) and the need for RRT and mortality, at least in an ICU setting [38]. Crucially however, the negative predictive value of this model was extremely high, with a more modest positive predictive value; not everyone with small creatinine changes subsequently experienced more severe AKI, but those with no creatinine changes and fewer risk factors were extremely unlikely to do so. Such approaches allow targeting of early AKI intervention and/or risk prevention.

### Urine Output Criteria

Although less often utilised in epidemiological research and clinical practice, current AKI definitions also include urine output criteria. The importance of oliguria is supported by epidemiological studies showing associations between AKI diagnosed on urine output criteria and adverse outcomes, although mainly in critical care settings [39]. Oliguria does not occur at the same time as changes in serum creatinine, so use of urine output criteria may identify AKI earlier but also may select a different (additional) patient cohort from those with changes in serum creatinine [40]. How best to employ urine output criteria for the diagnosis of AKI outside critical care requires further study, as does consideration of potential unintended consequences (e.g., iatrogenic urinary tract sepsis resulting from increased rates of catheterisation).

### Does Improved Diagnosis of AKI Matter?

International guidelines, including those from the National Institute for Health and Care Excellence [41], advocate that management of AKI is based around supportive care (correction and avoidance of hypovolaemia, prompt treatment of sepsis and shock, avoidance of medications that may cause or worsen AKI, appropriate investigation to determine aetiology, and timely referral of patients with need of specialist input), although the evidence to support the specific recommendations within guidelines is currently incomplete. Along with the lack of specific pharmacotherapies for AKI, this has led some to question the value of applying diagnostic criteria in clinical care. However, if the utilisation of the diagnostic criteria enables better recognition and improves early diagnosis, then this is a strong argument for their use. Whilst evidence for current AKI management strategies needs expansion, there are clear signals that failure to deliver a good standard of supportive care leads to poor outcomes; despite recent advances, we know that this still occurs commonly across a variety of health care systems [42–48]. Efforts to address standards of care that are below the minimum recommendation of currently acceptable practice should not be controversial, and if linked to data collection to measure their effect, they will add to the current evidence base. Testing new approaches is important, as there have been reports of both successes and failures of quality improvement initiatives [49–51]. The requirement for consistent measurement of incidence and outcomes becomes a further argument for adoption of standardised AKI diagnostic criteria. We are beginning to see more sophisticated measurement approaches—such as a national AKI registry, as proposed by the National Health Service (England) “Think Kidneys” programme [52]—and at the same time, it is vital that the causes and risk factors for AKI in LMICs are better studied to inform preventative and public health strategies [53].

### Diagnosis of AKI in LMICs

The challenges of applying current AKI criteria in LMICs are very different and much more fundamental. They include limited laboratory resources coupled with logistical issues, preventing timely serum creatinine concentration measurement or even measurement at all; lack of availability of previous creatinine results; financial constraints, meaning that some patients can’t afford serial creatinine tests (upon which current criteria are based); and late presentation of patients to health care services, thereby reducing opportunities for early detection and prevention. Work is accelerating to address this—for example, a recent description of key areas of need along with practical suggestions for quality improvement [54]. The 0by25 programme also discusses a number of strategies, such as educational campaigns (including the promotion of urine output criteria to diagnose AKI) and use of inexpensive point-of-care testing [11]. Even the latter may not be straightforward—for example, point-of-care creatinine equipment at present requires an electrical power source that may prevent use in certain contexts. It remains to be seen as to whether the current diagnostic criteria for AKI need refinement to improve suitability for settings that are far removed from the developed countries in which they were first conceived. The challenges of tackling AKI in LMICs extend to public health, resource, and organisational issues that at present must take priority.

## Future Directions and Developments

At present, we believe the advantages of using the current diagnostic criteria for AKI outweigh perceived disadvantages. However, serum creatinine concentration has a number of limitations as a biomarker of AKI, not least that it is affected by a number of factors other than renal function, the delay before it rises after renal injury, and that as a functional marker it does not provide information about the nature or aetiology of renal damage. These obvious deficiencies have driven extensive research activity to identify novel biomarkers for AKI, although none have yet found a place in clinical practice. There is increasing realisation that a “single-shot” diagnostic biomarker will not be identified, in part reflecting the heterogeneous nature of AKI. Future directions may include: better clinical targeting of biomarkers to phenotypes and/or aetiologies of AKI; the combination of functional and damage biomarkers; or a two-step process, the first highly sensitive but relatively nonspecific, followed by a second, more specific test to rule in the diagnosis [55,56]. Future advances in other diagnostic modalities such as imaging may also support biomarker development.

There are significant challenges to improving the recognition of AKI in resource-poor settings, and research is needed to not only to test the effectiveness of new approaches but also their feasibility and sustainability. Of relevance to all settings is the need to raise the awareness of AKI. This is not limited to health care workers but also must incorporate the public and patients, whose understanding of the role of kidneys and implications of kidney damage are currently low [11].

## Conclusion

AKI is a major challenge to health care providers and clinicians. Current diagnostic criteria bring opportunities to identify patients at higher risk of adverse outcomes as well as providing a standardised and evidence-based approach to AKI recognition but require clinical interpretation for individual patient management. Whilst approaches to AKI are hugely different between developed and developing countries, the principles of AKI detection underpin efforts to improve delivery of AKI care as well methods to measure its impact and outcomes.

## Abbreviations

AKI: acute kidney injury
CKD: chronic kidney disease
GFR: glomerular filtration rate
KDIGO: Kidney Disease: Improving Global Outcomes
LMICs: low- and middle-income countries
RRT: renal replacement therapy
